# Necklace‐embedded electrocardiogram for the detection and diagnosis of atrial fibrillation

**DOI:** 10.1002/clc.23580

**Published:** 2021-02-25

**Authors:** Onni E. Santala, Jukka A. Lipponen, Helena Jäntti, Tuomas T. Rissanen, Jari Halonen, Indrek Kolk, Hanna Pohjantähti‐Maaroos, Mika P. Tarvainen, Eemu‐Samuli Väliaho, Juha Hartikainen, Tero Martikainen

**Affiliations:** ^1^ School of Medicine University of Eastern Finland Kuopio Finland; ^2^ Department of Applied Physics University of Eastern Finland Kuopio Finland; ^3^ Center for Prehospital Emergency Care Kuopio University Hospital Kuopio Finland; ^4^ Heart Center North Karelia Central Hospital Joensuu Finland; ^5^ Heart Center Kuopio University Hospital Kuopio Finland; ^6^ Department of Clinical Physiology and Nuclear Medicine Kuopio University Hospital Kuopio Finland; ^7^ Department of Emergency Care Kuopio University Hospital Kuopio Finland

**Keywords:** Awario analysis service, arrhythmia, atrial fibrillation, ECG, stroke, Suunto Movesense

## Abstract

**Background:**

Atrial fibrillation (AF) is the major cause of stroke since approximately 25% of all strokes are of cardioembolic‐origin. The detection and diagnosis of AF are often challenging due to the asymptomatic and intermittent nature of AF.

**Hypothesis:**

A wearable electrocardiogram (ECG)‐device could increase the likelihood of AF detection. The aim of this study was to evaluate the feasibility and reliability of a novel, consumer‐grade, single‐lead ECG recording device (Necklace‐ECG) for screening, identifying and diagnosing of AF both by a cardiologist and automated AF‐detection algorithms.

**Methods:**

A thirty‐second ECG was recorded with the Necklace‐ECG device from two positions; between the palms (palm) and between the palm and the chest (chest). Simultaneously registered 3‐lead ECGs (Holter) served as a golden standard for the final rhythm diagnosis. Two cardiologists interpreted independently in a blinded fashion the Necklace‐ECG recordings from 145 patients (66 AF and 79 sinus rhythm, SR). In addition, the Necklace‐ECG recordings were analyzed with an automatic AF detection algorithm.

**Results:**

Two cardiologists diagnosed the correct rhythm of the interpretable Necklace‐ECG with a mean sensitivity of 97.2% and 99.1% (palm and chest, respectively) and specificity of 100% and 98.5%. The automatic arrhythmia algorithm detected the correct rhythm with a sensitivity of 94.7% and 98.3% (palm and chest) and specificity of 100% of the interpretable measurements.

**Conclusions:**

The novel Necklace‐ECG device is able to detect AF with high sensitivity and specificity as evaluated both by cardiologists and an automated AF‐detection algorithm. Thus, the wearable Necklace‐ECG is a new, promising method for AF screening. Clinical trial registration: Study was registered in the ClinicalTrials.gov database (NCT03753139).

## 1 INTRODUCTION

Atrial fibrillation (AF) is a rapidly growing global public health problem.^1^ At the population level, the lifetime risk for developing AF is around 25%.^2^ One in four middle‐aged adults in the EU and the United States will develop AF.^3^, ^4^ The most severe complication of AF is an embolic stroke; approximately 25% of all strokes are caused by AF.^5^ Stroke imposes a significant financial burden on the health care system, with a total cost of over €40 billion in both the European Union and the United States.^6^, ^7^ These costs will continue to rise as the population ages and lives longer. It has been claimed that two out every three AF related strokes can be prevented by the provision of appropriate oral anticoagulation therapy as long as AF is diagnosed early enough.^8^

New screening methods for AF in different scenarios are being intensively studied.^9^, ^10^, ^11^ Current guidelines state that a single‐lead electrocardiogram (ECG) tracing of ≥30 s or 12‐lead ECG analyzed by a physician is necessary to establish a definitive diagnosis of AF.^12^ Traditional invasive and non‐invasive methods for AF detection, such as ambulatory ECGs (Holter), loop recorders and prolonged ambulatory ECGs (mobile cardiovascular telemetry) are expensive and require interpretation by healthcare professionals.^13^

There are several commercial handheld ECG devices for AF screening, designed for consumer use.^14^, ^15^, ^16^, ^17^, ^18^, ^19^ These kinds of devices can represent a relatively cost‐efficient solution for AF screening, since many patients are motivated to monitor their own health.^15^, ^17^ However, to be effective, users must incorporate such devices into their everyday use. It is evident that device design plays a significant role in user adoption and therefore clever design can increase the usage of these devices over the long term.^20^ Many commercial health products can be too technical and complicated for the consumers in high‐risk groups, such as the elderly.^21^

Therefore, in this study, we evaluated the feasibility and accuracy of a novel ECG recording technique, a wearable Necklace‐ECG, for the detection of AF by (a) cardiologists and (b) an automated algorithm.

## 2 METHODS

### 2.1 Study design

The study was performed as a single‐center case–control study. Local Ethics Committee approved the study protocol (01/08/2018) and the study was registered in the ClinicalTrials.gov database (NCT03753139).

### 2.2 Study population

**FIGURE 1:**
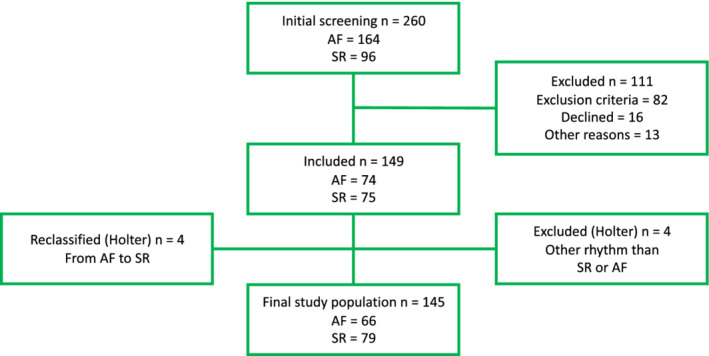
Study population flow chart. AF, atrial fibrillation; SR, sinus rhythm

A total of 260 patients was screened in our emergency department between November 2018–May 2019. The flowchart of the screening of study participants is presented in Figure 1. The study inclusion criteria were AF or sinus rhythm (SR) on a 12‐lead ECG recorded during the in‐hospital treatment period. The exclusion criteria were body mass index (BMI) ≥ 35 kg/m^2^, left bundle branch block or right bundle branch block, implanted cardiac pacemaker, a medical condition requiring immediate treatment and other rhythm than AF or SR. In the initial screening, 111 patients were excluded; 82 met the exclusion criteria, 16 patients declined to participate and 13 were excluded for other reasons (e.g., oncoming cardioversion, medical examinations, discharge from the hospital). In addition, the final rhythm classification made from Holter ECG recording reclassified four patients from the AF group to the SR group and revealed four patients with a rhythm other than SR or AF. Thus, the latter four patients were excluded from the study.

Finally, 65 patients were assigned to the AF group and 75 patients to the SR group. Of the AF patients, 26 had recent‐onset AF (<48 hours) and 40 patients late AF (≥48 hours). A written information and the opportunity to ask questions about the study were given to all study participants. All participants provided written informed consent to participate in the study.

### 2.3 Necklace‐ECG recording

**FIGURE 2:**
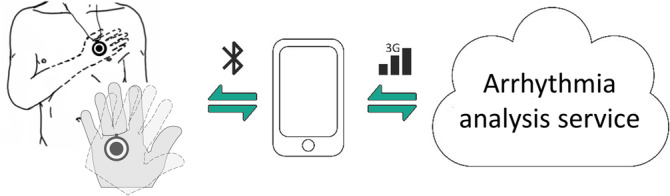
Schematic presentation of Necklace‐electrocardiogram enabled automatic arrhythmia analysis

All study subjects performed an at least thirty‐second self‐performed ECG recordings in the sitting, half‐sitting or lying down positions using two measurement positions with a single‐lead Necklace‐embedded ECG recorder (Including Movesense ECG‐sensor, Suunto, Vantaa, Finland, Necklace‐ECG, Figure 2 and Figure S1). First, the subjects were holding the recorder between the palms of their hands (palm) and secondly, between the chest and the right palm (chest), both measurement positions simulate lead I ECG. A maximum of three measuring attempts per position were allowed.

### 2.4 Continuous ECG recording

The final rhythm classification was based on simultaneously recorded 3‐lead ECG (Holter, Faros 360, Bittium, Oulu, Finland) in the sitting, half‐sitting or lying down positions. Two experienced cardiologists interpreted the ECG recordings independently. In case of disagreement, they reviewed the ECG recording together to confirm the final rhythm analysis.

The clinical characteristics of the patients including age, gender, height, weight, BMI, concomitant diseases and medications were collected by interview and confirmed/complemented from the medical records.

### 2.5 Necklace‐ECG analysis

Two experienced cardiologists interpreted the Necklace‐ECG recordings independently in a random order and blinded to the Holter ECG recordings and the initial 12‐lead ECG. In case of disagreement, they reviewed the ECG recording together to confirm the final rhythm analysis. They divided the rhythm of the Necklace‐ECG recordings into four categories: sinus rhythm, atrial fibrillation, other and non‐interpretable. Accordingly, the quality of Necklace‐ECG was classified into four categories: high, average, poor and failure. In addition, the possibility of detecting P‐waves from the ECG strips showing SR (yes/no) was assessed.

A commercial arrhythmia analysis service (Awario, Heart2Save, Kuopio, Finland) was used to test the accuracy of automatic AF‐screening from the Necklace‐ECG recording. The Necklace‐ECG data was transferred to the cloud‐based analysis service via a mobile phone application (Figure 2). An automatic arrhythmia algorithm classified the Necklace‐ECG strips into three categories: sinus rhythm, atrial fibrillation or non‐interpretable.

### 2.6 Statistical analysis

The sample size was estimated to be 150 observations with an assumed sensitivity of 95% with the method and with a 3.5% margin error, such that the sensitivity parameter with 95% certainty lay between 91.5% and 98.5%. The AF and SR groups were compared using *t*‐test for continuous variables and χ^2^ test or Fisher's exact tests for dichotomous variables. The Kappa‐coefficient was calculated to measure the level of consensus between the “golden standard” (rhythm diagnosis from the Holter ECG recordings) and the cardiologists AF‐diagnosis and algorithm AF‐detections from the Necklace‐ECG. Additionally, the sensitivity and specificity of AF detection were determined for the cardiologists and automatic arrhythmia detection algorithm. All significance tests were two‐tailed and *p* ≤ .05 was considered statistically significant. The data was analyzed using IBM SPSS statistics software version 25.

## 3 RESULTS

### 3.1 Clinical characteristics

The consensus rhythm of two experienced cardiologists from 3‐lead Holter ECG was used as “golden standard” for the rhythm analysis. The final study population consisted of 66 AF patients and 79 SR patients, (Figure 1). In the AF group, 26 (39%) patients had recent‐onset AF (duration < 48 hours) and 40 (61%) patients had late AF (duration ≥ 48 hours). Patient demographics are presented in Table 1. AF patients were older than the SR patients (72.7 ± 14.1 vs. 61.5 ± 18.1 years, *p* < .001). AF patients also presented more often with a history of paroxysmal AF (*p* < .001), asymptomatic AF (*p* < .001), congestive heart failure (*p* < .001) and were more often being treated with anticoagulation (*p* < .001), beta‐blocker (*p* < .001) and digoxin (*p* < 0.05) therapy. AF patients also reported more often respiratory distress (*p* < .001), compared to the SR group.

**TABLE 1:** Patient demographics

	SR group (*N* = 79)	AF group (*N* = 66)	Significance (2‐sided)
Characteristics			
Age (years)	61.5 ± 18.1	72.7 ± 14.1	<.001
BMI	26.7 ± 4.3	27.2 ± 4.7	0.484
Male gender	37 (46.8%)	29 (43.9%)	0.727
Medical history			
Earlier AF episode	11 (13.9%)	44 (66.7%)	<.001
Asymptomatic AF (currently or in the history)	7 (8.9%)	22 (33.3%)	<.001
Coronary heart disease	15 (19.0%)	20 (30.3%)	0.113
Diabetes mellitus	14 (17.7%)	8 (12.1%)	0.349
Hypertension	47 (59.5%)	48 (72.7%)	0.095
Congestive heart disease	3 (3.8%)	25 (37.9%)	<.001
Previous heart surgery	4 (5.1%)	8 (12.1%)	0.125
Structural heart defect	4 (5.1%)	2 (3.0%)	0.689
Medication			
Anticoagulation therapy	20 (25.0%)	62 (81.8%)	<.001
Beta‐blocker	32 (40.5%)	50 (75.8%)	<.001
Digoxin	1 (1.3%)	6 (9.1%)	0.047
Other anti‐arrhythmia medication	1 (1.3%)	1 (1.5%)	1.00
Symptoms prior to hospital admission			
Fatigue	50 (63.3%)	50 (75.8%)	0.106
General state decline	44 (55.7%)	47 (71.2%)	0.054
Heart palpitations	27 (34.2%)	29 (43.9%)	0.229
Respiratory distress	21 (26.6%)	38 (57.6%)	<.001
Chest pains	23 (29.1%)	18 (27.3%)	0.806
Other symptoms	34 (43.0%)	17 (25.8%)	0.030

### 3.2 Diagnosing AF by Necklace‐ECG

Necklace‐ECG measurements produced an interpretable ECG‐strip in 88.6% (mean of the two cardiologists) patients from the palm and 83.75% from the chest. The quality of ECG recordings was estimated as high or average in 62.4% and poor in 26.2% of the palm measurement. Similarly, for the chest measurements, in 63.4% of ECGs, the quality was estimated as high or average and 20.3% as poor. In the SR group, P‐waves were identified in 94.3% of the interpretable palm measurements and in 85.5% of the interpretable chest measurements. Representative examples of Necklace‐ECG measurements are presented in Figure S2.

The consensus of the two cardiologists regarding AF‐diagnosis from the Holter and Necklace ECGs are presented in Table 2. Based on the interpretable Necklace‐ECG recordings, AF was diagnosed with a mean sensitivity of 97.2% using the palm and 99.1% using the chest measurements. The mean specificity was 100% and 98.5% using the palm and the chest measurements. For the cardiologists interpretation of the Necklace‐ECG, the mean kappa's coefficient with the cardiologists diagnosis from Holter ECG was almost perfect (κ = 0.975) using both the palm and the chest measurements, see Table 2. The two cardiologists agreed on the diagnosis of the Necklace‐ECG in 123 out of 124 cases giving a kappa value of 0.983 for palm measurements (21 non‐interpretable cases) and in 108 out of 109 cases giving a kappa value of 0.982 for chest measurements (36 non‐interpretable cases).

**TABLE 2:** Atrial fibrillation (AF)‐diagnosis of cardiologist‐interpreted Necklace‐electrocardiogram (ECG) recordings compared to two cardiologists' consensus of rhythm from Holter ECGs

	Cardiologist 1 Necklace‐ECG			Cardiologist 2 Necklace‐ECG		
Holter ECG diagnosis	SR	AF	Non‐interpretable	SR	AF	Non‐interpretable
Palms						
SR	**77** (98%)	**0** (0%)	2 (2%)	**71** (90%)	**0** (0%)	8 (10%)
AF	**1** (2%)	**54** (82%)	11 (17%)	**2** (3%)	**52** (79%)	12 (18%)
**Specificity: 100%** (100%/100% Doc1/Doc2), **Sensitivity: 97.2%** (98.2%/96.3%) and **Kappa 0.975** (0.984/0.967) from interpretable (bold) palm measurements.						
Chest						
SR	**75** (95%)	**1** (1%)	3 (4%)	**56** (71%)	**1** (1%)	22 (28%)
AF	**1** (2%)	**54** (81%)	11 (16%)	**0** (0%)	**55** (83%)	11 (17%)
**Specificity: 98.5** (98.7%/98.2% Doc1/Doc2) and **Sensitivity: 99.1** (98.2%/100%) and **Kappa 0.975** (0.969/0.982) from interpretable (bold) chest measurements.						

### 3.3 AF‐screening with an automatic algorithm

The automatic arrhythmia algorithm found 93.1% and 92.4% (palm and chest, respectively) of ECG recordings interpretable. The AF detection sensitivity was 94.7% (palm) 98.3% (chest) and specificity 100% (palm and chest) from the interpretable measurements. For the automatic arrhythmia algorithm interpretation of the Necklace‐ECG, the kappa's coefficient with the cardiologists diagnosis from Holter ECG was almost perfect (palm κ = 0.954/chest κ = 0.984) using both the palm and the chest measurements, see Table 3.

**TABLE 3:** AF‐detections from Necklace‐electrocardiogram (ECG) recordings interpreted by the arrhythmia analysis algorithm as compared to two cardiologists' consensus from Holter ECGs

	Algorithm palms			Algorithm chest		
Holter ECG diagnosis	SR	AF	Non‐interpretable	SR	AF	Non‐interpretable
SR	**78** (99%)	**0** (0%)	1 (1%)	**75** (95%)	**0** (0%)	4 (5%)
AF	**3** (5%)	**54** (82%)	9 (14%)	**1** (2%)	**58** (88%)	7 (10%)
**Specificity: 100%/100%** (palm/chest), **Sensitivity: 94.7%/98.3%** and **Kappa: 0.954/0.984** from interpretable (bold) measurements						

## 4 DISCUSSION

The main finding of our study was that the wearable Necklace‐ECG device was able to produce ECG recordings with high quality for the detection and diagnosis of AF and SR with high sensitivity and specificity as evaluated by two cardiologists as well as by an automated arrhythmia detection algorithm.

The diagnosis of AF always requires confirmation from an ECG recording.^12^ There are many different methods available in the hospital for diagnosing atrial fibrillation. Clinical non‐invasive screening devices include continuous ECG telemetry, ambulatory ECG (Holter), patient‐triggered event recorders and prolonged ambulatory ECG (mobile cardiovascular telemetry). Invasive screening devices include implantable loop recorders, pacemakers and implantable cardioverter defibrillators.^13^ However, these methods are invasive and expensive and thus, they are not suitable for large scale AF screening.^13^ Therefore, novel methods for AF detection are needed, especially as the incidence of AF will increase due to the aging of populations. One cost‐effective solution for AF screening could be an easily accessible “handheld ECG” device designed for consumer use, such as the Necklace‐ECG^14^, ^15^, ^16^, ^17^

ECG‐based handheld devices have been evaluated for AF‐detection, either interpreted by physicians or by using automated algorithms.^14^, ^15^, ^16^, ^17^ In these studies, the handheld devices have been compared with the traditional 12‐ or 6‐lead ECG registration with inconclusive results. In terms of signal quality, between 0.8% to 13% of handheld ECG recordings were reported to be inadequate for rhythm diagnosis as interpreted by physicians^14^, ^15^, ^17^, ^22^, ^23^ and 3.9%–33.7% as uncapable of being interpreted by an automated algorithm.^14^, ^17^, ^22^, ^23^ Our study is in line with these earlier reports; Necklace‐ECG recordings were judged as non‐interpretable in 9.0%–23% of all patients and in 16%–18% of patients with AF. Somewhat surprisingly, the quality of ECG recordings was sufficient for AF detection more often when interpreted by the automated algorithm than when interpreted by the cardiologist. Only 6%–8% of the Necklace‐ECG recording were deemed non‐interpretable with the automatic arrhythmia algorithm. One reason for the inadequate quality was that in cases where there was any uncertainty, the cardiologists were advised to label the recordings as non‐interpretable.

In our study, the Necklace‐ECG yielded a sensitivity of 95%–100% and specificity of 98%–100% with either physician or automatic arrhythmia detection algorithms. This also corroborates earlier studies using handheld devices; these have reported AF‐detection sensitivities ranging from 77% to 100% and specificities from 80% to 96% when interpreted by the physicians and sensitivities of 55%–100% and specificities of 84%–98% when the interpretation was performed using automated algorithms.^14^, ^15^, ^16^, ^17^, ^23^

The abundance of health information devices and the large consumer population show an increasing interest in self‐monitoring. In addition, it has been claimed that patients with chronic diseases are interested in monitoring their health.^24^ Handheld single‐lead ECG devices have been proven to be cost effective in AF screening in the following settings: hospital patients,^15^ general study population of 75–76‐year‐olds^25^ and patients with a recent ischemic stroke.^26^ Handheld ECG devices could enable repetitive rhythm monitoring over the long term and thus improve the probability of AF detection. However, to be effective, handheld ECG devices must be accurate and users must adopt the devices into their everyday routines.

We have shown that with this all‐time wearable Necklace‐ECG, AF can be detected and diagnosed with high accuracy. The wearable Necklace‐ECG could provide a user‐friendly alternative to AF screening, especially for the elderly, as it is wearable, and the ECG recording is easy to perform. Also, some users do not want to be identified as a cardiac patient. For them, the necklace is an elegant way to monitor arrhythmias. In cardiac rhythm monitoring, the Necklace‐ECG allows the patient to perform repeated screening or symptom‐based, such as palpitation or pre‐ or post‐syncope, recordings to identify undiagnosed AF. After the measurement, the mobile application (Figure 2) displays the results of the automatic arrhythmia analysis and the ECG report to the patient. His/her physician confirms the arrhythmia diagnosis based on the ECG report, which can be accessed from the patient's application or directly from the arrhythmia analysis service. The Necklace‐ECG provides a novel method for accurate automatic AF screening and physician‐confirmed diagnosis. Further studies are needed to analyze the hypothesized positive effect of design on user adoption and the efficacy of the device in outpatient and home screening for AF.

There are some limitations in this study. First, the patients in the trial were trained to perform the Necklace‐ECG measurements and the measurement conditions were optimal with a resting patient in the sitting, half‐sitting or lying down position. However, in real life, the inappropriate use or movement of hands, may increase the number of non‐interpretable measurements. Second, the study patients were recruited from the hospital emergency room where patients are often in poor condition and need a lot of care. Therefore, the number of excluded patients was relatively high, 32 patients requiring immediate treatment were excluded. Third, severe obesity decreases ECG signal amplitude, and bundle branch block would likely result in more noninterpretable recordings. For these reasons, these groups have been excluded from this study. Further studies in these subgroups are needed. In addition, a handheld ECG recorder needs activation by the subject, thus, the detection of short, asymptomatic AF episodes remains a challenge.

## 5 CONCLUSIONS

The Necklace‐ECG recorder produces ECG signal with sufficient quality for the detection of AF with good sensitivity and specificity as evaluated both by two cardiologists and an automated arrhythmia detection algorithm. Thus, repeated ECG recording with the Necklace‐ECG and an automatic arrhythmia detection algorithm might be useful in the screening of AF in a high‐risk population. This wearable measuring device could provide a new and easy method for screening, identifying and diagnosing AF.

## CONFLICT OF INTEREST

Jukka A. Lipponen, Tuomas T. Rissanen, Tero Martikainen, Helena Jäntti, Jari Halonen and Mika P. Tarvainen are shareholders of a company (Heart2Save) that designs ECG‐based software for medical equipment. Kuoppa P, Jukka A. Lipponen, Mika P. Tarvainen and Helena Jäntti report personal fees from Heart2Save. There are no other conflicts of interest to declare.

## Supporting information


**Figure S1.** Necklace‐embedded ECG recorder.

**Figure S2.** Representative examples of Necklace‐ECG recordings, chest, palms and lead I from Holter ECG.
