# Supplementary figures and images for: Necklace‐embedded electrocardiogram for the detection and diagnosis of atrial fibrillation
Source: Clin Cardiol. 2021 Feb 25;44(5):620–6. doi: 10.1002/clc.23580 (PMC8119818; doi:10.1002/clc.23580)

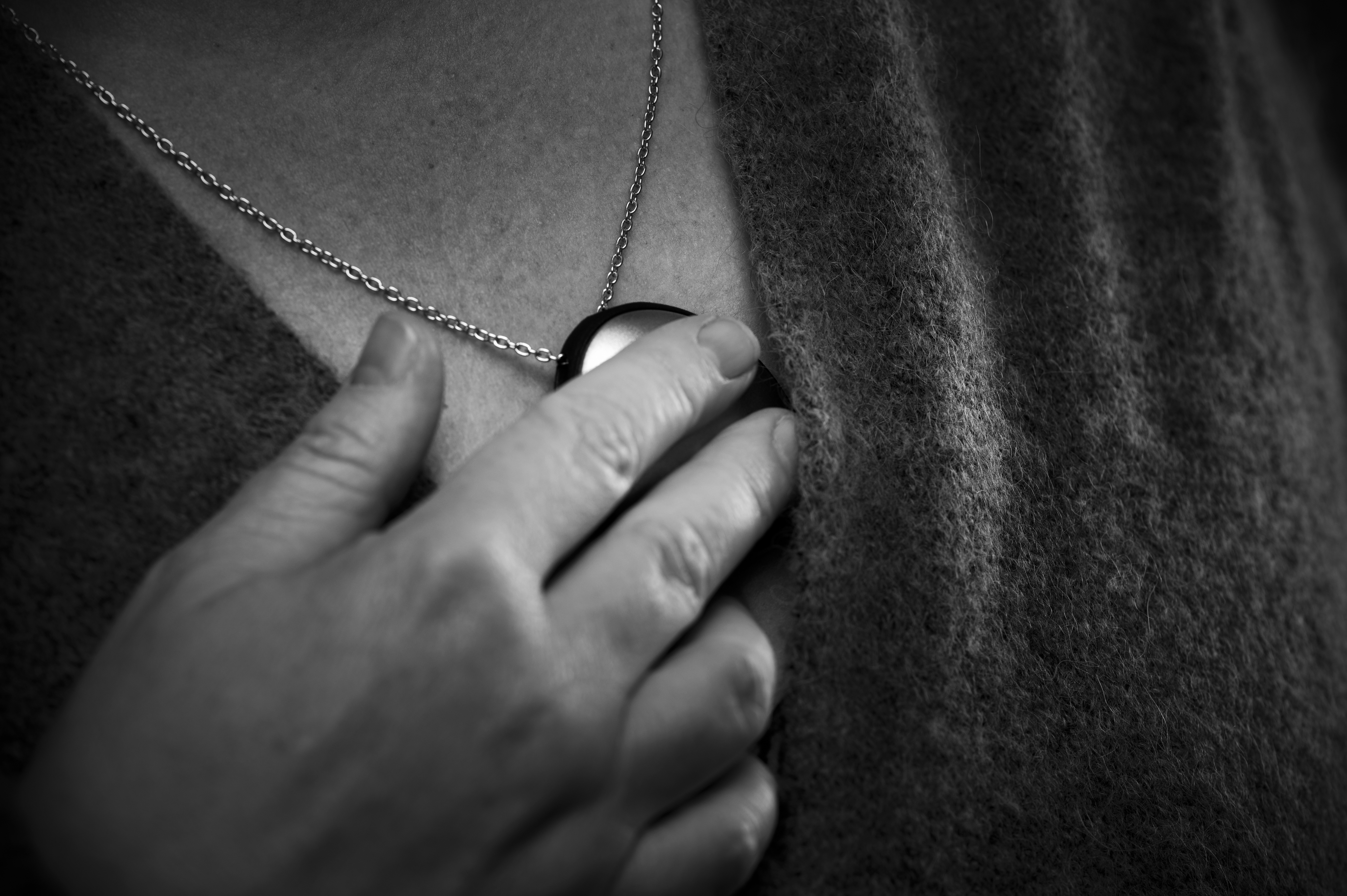

Supplement: Supplementary file 1 — Figure S1. Necklace‐embedded ECG recorder. [file CLC-44-620-s001.jpg]

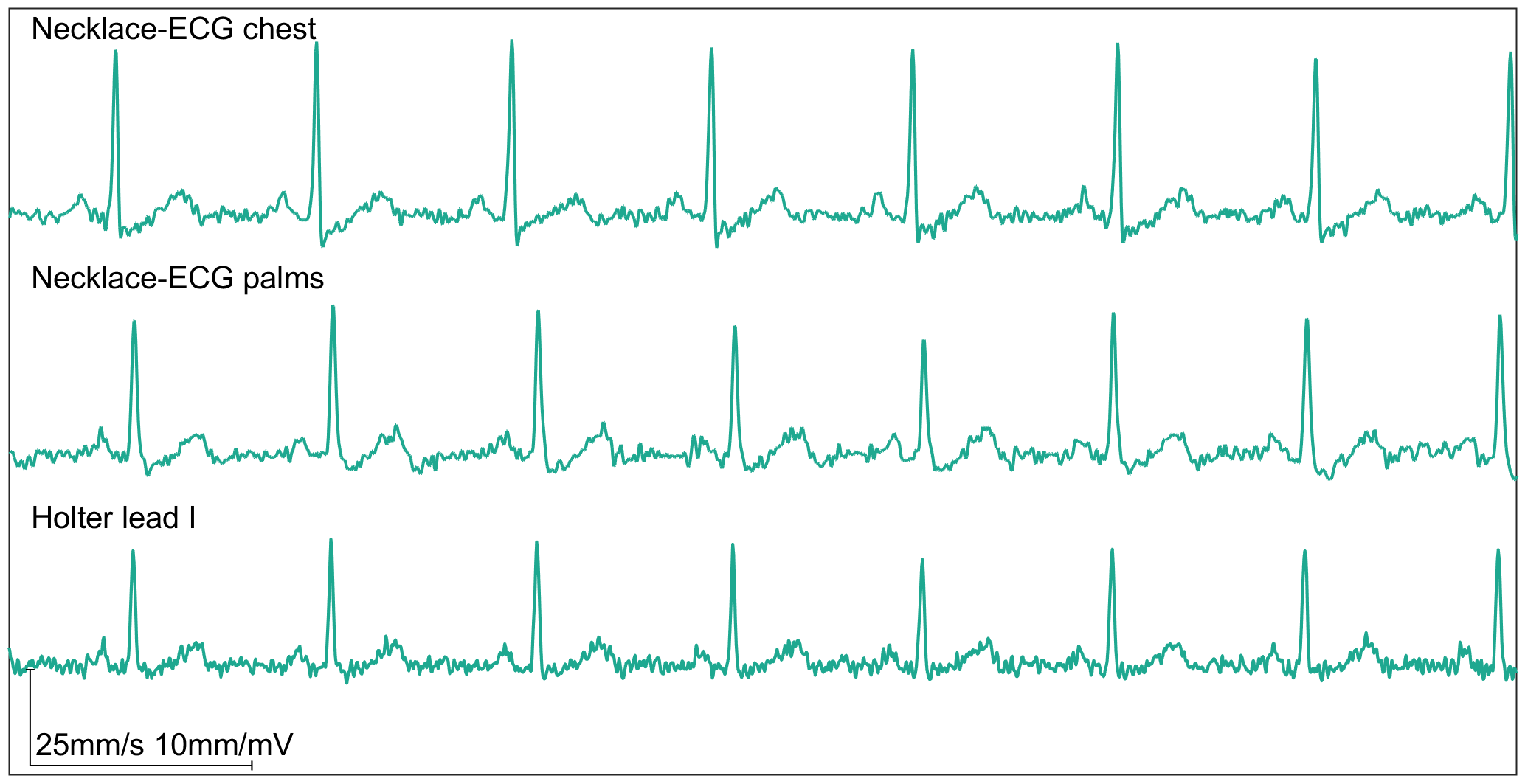

Supplement: Supplementary file 2 — Figure S2. Representative examples of Necklace‐ECG recordings, chest, palms and lead I from Holter ECG. [file CLC-44-620-s002.tiff]
